# Health expenditure, governance and SDG3 nexus: a longitudinal analysis in BRICS economies

**DOI:** 10.1186/s12992-025-01113-8

**Published:** 2025-04-09

**Authors:** Md. Mominur Rahman, Tasneem Islam Dyuti, Mohammad Tareque, Mohammed Alnour

**Affiliations:** 1 Bangladesh Institute of Governance and Management (BIGM), Dhaka, Bangladesh; 2 Department of Economics, Boston University, Boston, MA USA; 3 Faculty of Economics, Social and Environmental Studies, University of Medical Sciences & Technology, Khartoum, Sudan

**Keywords:** Health expenditures, Governance, SDG3, Quality institutions

## Abstract

**Background:**

Achieving Sustainable Development Goal 3 (SDG3): good health and well-being, requires significant health investments and effective governance. While many studies explored the influence of health expenditure and governance, little is known about how different levels of governance affect the relationship between health expenditure and SDG3 in a globalised world. Thus, this study aims to fill that gap by examining the marginal effects of health expenditure on SDG3 under varying levels of governance in BRICS economies.

**Methods:**

This study uses quantitative data spanning a panel of 2000–2023 years. Governance is measured using worldwide governance indicators, while health spending is represented by current health costs, government health costs, and private health costs from the World Development Indicators. Data on SDG3 comes from the SDG Index. Cross-sectional dependency, stationarity and cointegration tests are employed to choose appropriate panel data models. The final results are obtained using Fully Modified OLS, while System GMM is used to address issues like endogeneity, autocorrelation, instrumentation, and causality. To ensure the results are reliable, the study also tests alternative measures of governance.

**Results:**

1% increase in current and government health spending improves SDG3 by 3.92% and 2.86%, respectively, while a 1% rise in private health spending reduces it by 0.677%. This negative impact in BRICS nations is likely due to market failures in private healthcare, where profit-driven models limit access and efficiency. The positive impact of current and government health expenditure on health outcomes is comparatively weaker at lower levels of governance but private health expenditure and SDG3 are weakening by governance at different levels which is indicative of inefficiencies in resource allocation and implementation. This study supports institutional theory, which states that strong governance improves the effectiveness of public health spending, leading to better health outcomes. The study highlights how the geopolitical prominence of governance frameworks interacts to optimise the benefits of health investments, demonstrating their role as leaders in advancing global health initiatives. Thus, policymakers need an integrated approach in health investments with institutional reforms in achieving health outcomes more effectively as good governance significantly amplifies the relationship.

**Conclusions:**

This study highlights that governance plays a key role in improving the impact of health spending on SDG3. Strong governance boosts the benefits of public health expenditure and limits the negative effects of private health expenditure. Thus, the findings stress the importance of effective governance in enhancing health outcomes in BRICS economies.

## Introduction

The global focus on health expenditure and the attainment of Sustainable Development Goal 3 (SDG3) is imperative for addressing the pressing health challenges faced by emerging nations [1, 2]. SDG3 aims to ensure healthy lives and promote well-being for all individuals, of all ages, by 2030 [3]. Given the rising burden of diseases, health inequities, and the increasing costs of healthcare, it is crucial to allocate adequate resources to health systems [4, 5]. Effective health expenditure not only improves access to essential healthcare services but also enhances overall health outcomes by addressing preventable diseases and ensuring equitable care [6]. As health systems worldwide grapple with these challenges, understanding the relationship between health expenditure and SDG3 becomes vital for formulating policies that can achieve meaningful progress in global health. Good governance plays a pivotal role in shaping health outcomes and is therefore a critical consideration in health policy and expenditure [7]. Governance refers to the structures, processes, and practices through which institutions are managed and decisions are made [8]. In the context of health systems, effective governance involves transparent decision-making, accountability, and efficient resource management [9]. It ensures that health expenditures are used effectively, policies are implemented fairly, and health services are delivered equitably [2, 10, 11]. Governance not only influences the efficiency and impact of health spending but also affects public trust and the overall effectiveness of health interventions [12]. Therefore, integrating governance considerations into health policy is essential for achieving sustainable health improvements.

Empirical research on the relationship between health expenditure and health outcomes has produced varied findings, with many studies highlighting a positive correlation, particularly in developing nations. Adu [13] and Rahman et al. [14] found that increased public health spending leads to improved health indicators, such as reduced mortality rates and longer life expectancy. Similar results were observed in East Africa [15], and Sub-Saharan Africa [16] reinforcing the importance of public investment in health. However, other studies emphasise the complexity of this relationship, noting that public health expenditure does not always translate into better health outcomes due to inefficiencies in healthcare systems [17, 18]. Additionally, Nixon and Ulmann [19] argue that multiple factors influence health outcomes beyond financial investments, while Sibanda et al. [20] highlight the critical role of out-of-pocket expenditures in mitigating healthcare gaps. These mixed findings indicate that the effectiveness of health spending is contingent on several factors, necessitating further investigation into contextual determinants such as governance quality.

Despite extensive research, the literature exhibits notable gaps, particularly concerning the BRICS nations, where health financing structures and governance models differ significantly. Existing studies, such as those by Sahoo et al. [21] and Romaniuk et al. [22], provide valuable insights into health system performance within BRICS but fail to examine how health expenditure contributes to SDG3 targets across these nations. Moreover, Rad et al. [23] highlight variations in public and private health expenditures across income groups but do not explicitly address the BRICS context. A critical oversight in the literature is the role of governance in shaping health outcomes, as governance quality can influence the efficiency and impact of health investments [7, 24]. Given the diverse governance frameworks within BRICS, ranging from Brazil’s democratic system to China’s centralised model [25], this study aims to fill this gap by analysing the marginal interactive role of governance in the health expenditure-health outcomes nexus. Furthermore, while prior research has acknowledged governance as a determinant of health policy effectiveness [26], there is a lack of empirical evidence on the marginal effects of health expenditure at different governance levels within BRICS, which our study seeks to address.

In an increasingly interconnected world, globalisation plays a pivotal role in shaping health systems by influencing economic policies, healthcare financing, technological advancements, and the flow of information across borders. The integration of advanced technologies in healthcare, such as electronic health records and telehealth, has transformed service delivery and improved efficiency, thereby addressing the challenges posed by globalisation [27, 28]. Furthermore, globalisation has heightened the urgency for countries to adopt cohesive strategies to address transnational health challenges, such as pandemics, non-communicable diseases, and health inequities. The COVID-19 pandemic exemplified how interconnectedness can exacerbate health crises, necessitating coordinated global responses [29]. Achieving SDG3 requires not only adequate health expenditure but also the integration of governance frameworks that ensure the resources are utilised effectively and equitably [30, 31]. The essence of governance in health development is crucial, as it encompasses the mechanisms through which health systems can be held accountable and responsive to the needs of diverse populations [32]. Globalisation magnifies the importance of robust health systems, efficient resource allocation, and governance mechanisms to address cross-border health challenges and disparities [33].

Institutional theory provides a valuable framework for understanding the relationship between health expenditure, governance, and health outcomes [34–37]. According to institutional theory, organisations and their practices are shaped by the institutional contexts in which they operate, including formal rules, norms, and cultural expectations [38]. In the realm of health systems, institutional theory suggests that both health expenditure and governance are influenced by broader institutional structures and processes. This theory helps explain why different countries with similar health expenditures might achieve varying health outcomes, depending on their governance quality and institutional settings [39]. Similarly, Rajkumar and Swaroop [40] demonstrated that governance quality moderates the impact of public health spending on health indicators, with better-governed countries achieving superior outcomes. Also, Filmer and Pritchett [41] stated that the relationship between health expenditure and health outcomes is often weak in low-governance settings, where institutional inefficiencies undermine the potential benefits of such spending. Therefore, The studies of Rajkumar and Swaroop [40] and Filmer and Pritchett [41] support the institutional theory regarding the interactive role of governance. By applying institutional theory, it is better to understand how governance frameworks and institutional contexts impact the effectiveness of health expenditures and contribute to achieving SDG3.

Despite the growing body of literature on health expenditures and governance, existing studies have predominantly focused on areas such as the direct impact of health spending on health outcomes or the general relationship between governance and health system performance [2, 4, 8, 9, 26, 42, 43]. This study is different from existing studies where the interaction effects between health expenditure and governance in influencing health outcomes are limited. The marginal interactive effects of governance on the relationship between health expenditure and health outcomes need to be investigated in the BRICS context. Health expenditures, encompassing both public and private spending, are crucial for improving healthcare systems and achieving SDG3 targets [26, 44]. However, the mere allocation of funds is not sufficient; the efficiency and effectiveness of these expenditures are significantly influenced by good governance [2, 11]. Good governance can enhance the impact of health expenditures, leading to better health outcomes [43]. Therefore, understanding how governance marginally interacts with the relationship between health expenditures and SDG3 is essential for crafting informed policy recommendations. This study aims to fill a critical gap in the existing literature by examining the interaction effects between health expenditure and governance on health outcomes, specifically within the context of BRICS economies. Therefore, the study will reveal how varied levels of governance like weaker, average, or better, lead to the marginal effect of health expenditure on SDG3 in emerging economies. Particularly the following pertinent questions are to be answered: what is the impact of health expenditure on attainment of SDG3? And what is the moderating influence of governance in the relationship between health expenditure and attainment of SDG3 in BRICS countries?

## Literature review

### Health expenditure and SDG3

Health expenditure is a crucial factor in attaining Sustainable Development Goal 3 (SDG3), which seeks to guarantee healthy lives and enhance well-being for individuals of all ages. Enhanced health expenditure is frequently linked to better health outcomes, including decreased death rates and extended life expectancy, especially in upper-middle-income nations, where enough per capita investment is crucial to achieving SDG3 objectives [45, 46]. This relationship is complex and contingent upon context. Insufficient health financing in low-income nations, exemplified by the Democratic Republic of Congo, where per capita health expenditure significantly lags behind necessary thresholds, results in adverse health outcomes, heightened morbidity, and enduring systemic costs [47]. Challenges such as dependence on out-of-pocket expenses, staff deficiencies, and inadequate training further diminish the efficacy of health expenditures, intensifying health inequities and restricting access to vital services [48, 49]. In contrast, in nations with sufficient health financing and financial protection systems, health expenditures substantially advance the achievement of universal health coverage (UHC), a crucial sub-target of SDG3, by alleviating the financial strain of catastrophic health costs [50]. To optimise the efficacy of health investments, it is essential to rectify systemic inefficiencies, guarantee equitable resource distribution, and enhance health system capacity, integrating augmented financing with strategic reforms to efficiently attain SDG3.

Health expenditure consists of multiple components, each exerting unique effects on healthcare outcomes. Current health expenditure, expressed as a proportion of GDP, functions as a comprehensive measurement of a nation’s entire dedication to healthcare. The effectiveness of health outcome improvement is substantially affected by governance quality since superior governance facilitates more efficient resource allocation and usage [9]. Domestic government health expenditure signifies direct governmental investment in healthcare services and infrastructure, frequently serving a vital function in guaranteeing fair access to critical healthcare, particularly in low- and middle-income nations. Conversely, domestic private health expenditure encompasses out-of-pocket expenditures and contributions from the private sector. Although private expenditure might enhance state investment in healthcare, overdependence on out-of-pocket expenses may create financial obstacles for vulnerable groups, worsening health inequities [9, 51, 52]. Therefore, comprehending the unexamined relationship between health expenditure and SDG3 is essential for maximizing health investments and enhancing health outcomes in BRICS countries. Empirical research endorsing institutional theory indicates that effectively controlled health systems enhance public health expenditure, resulting in better health outcomes and universal health coverage [9, 50]. Inefficient governance and structural flaws undermine the relationship between health expenditure and SDG3, particularly in low-income countries where insufficient funding leads to adverse health outcomes [30, 48]. Furthermore, dependence on out-of-pocket health expenditures without sufficient institutional protections constrains fair access, hence corroborating the institutional theory’s claim that governance frameworks influence the efficacy of health investments [51, 52].

### Governance and SDG3

Good governance is crucial for executing health policies, promoting accountability, and guaranteeing resource distribution, all of which are fundamental for attaining SDG3. Effective governance frameworks bolster health system resilience, foster transparency, and cultivate collaborations to tackle global health challenges, particularly emergencies [53, 54]. Moreover, governance affects health finance strategies, as sufficient government expenditure on health and strategic investments are essential for enhancing health outcomes, especially in developing economies [55, 56]. Poor governance, marked by inefficiencies, corruption, and disregard for equality, impairs health systems and intensifies inequities in healthcare access and outcomes [57, 58]. Factors such as gender inequality and insufficient resource allocation hinder advancement toward SDG3 [59]. The COVID-19 pandemic revealed deficiencies in governance systems, emphasizing the necessity for resilient frameworks to coordinate responses and provide equitable healthcare access [60]. Consequently, attaining SDG3 necessitates enhancing governance frameworks, promoting collaboration, and emphasizing equity to optimise health outcomes and system efficacy.

The relationship between good governance and health outcomes has been a significant focus of recent research, revealing the critical role governance plays in influencing health expenditure and outcomes across different regions [8, 10, 11, 24, 26, 61, 62]. Farag et al. [42] underscore that good governance is instrumental in ensuring efficient health spending, which positively impacts health outcomes. In contrast, Banik et al. [4] explore the connection between governance and health expenditure in South Asian nations, finding that the impact of governance on health spending is relatively modest. Hilaire [24] provides empirical evidence from Africa, indicating that effective governance significantly enhances the effectiveness of public health expenditure. Similarly, Ahmad and Hasan [63] analyse the interplay between health expenditure, governance, and health outcomes in Malaysia, finding that good governance helps reduce corruption and promotes more efficient health spending, which in turn positively impacts health outcomes. In BRICS economies, the relationship between good governance and SDG3 is under-explored necessitating the investigation. Empirical studies affirm the institutional theory by demonstrating that governance quality significantly influences health expenditure effectiveness and health outcomes [24, 42]. Research from Africa and Malaysia highlights that strong governance structures reduce corruption, improve resource allocation, and enhance public health spending efficiency, leading to better health outcomes [24, 63]. However, findings from South Asia suggest that governance’s impact on health expenditure may be limited in certain contexts, underscoring the need for further investigation, particularly in BRICS economies [4].

### Interaction effect of governance

Good governance guarantees the optimal allocation of health expenditures, so optimizing their influence on public health. Research indicates that strong governance frameworks can improve resource utilisation efficiency, allocating funding to effective health initiatives, such as decreasing maternal death rates [17, 64]. Governance affects the prioritizing of health expenditures, as demonstrated by Raghupathi and Raghupathi [65], who contend that public health investment enhances economic performance in well-governed contexts. In contrast, inadequate governance frameworks can result in suboptimal resource allocation, as demonstrated by variable health outcomes in targeted disease expenditures, including HIV/AIDS and tuberculosis [66]. This highlights the significance of governance in enhancing the connection between health expenditure and SDG3 objectives.

Additionally, governance plays a vital role in implementing financial protection mechanisms that support equitable access to healthcare. Effective governance facilitates the development and execution of micro-health insurance schemes, which are particularly beneficial in low-income settings by enhancing financial protection and access to care [67]. This is crucial for achieving SDG3’s overarching goal of promoting well-being for all. Onofrei et al. [52] further emphasise the interplay between governance, health expenditure, and public health outcomes, showing that governance quality is a key determinant of healthcare system performance in developing countries. Therefore, improving governance structures and processes is essential for strengthening the relationship between health expenditure and SDG3, enabling sustainable progress in health outcomes. Particularly, investigating the relationship between health expenditure and SDG3 within the BRICS context is essential, particularly because the moderating role of good governance in this relationship remains unexplored [4, 11, 42, 61, 62, 68]. Each BRICS country exhibits unique health funding structures and governance practices, influencing the allocation and utilisation of resources. For example, while Brazil and China invest significantly in public health, India and South Africa grapple with lower government spending and substantial out-of-pocket costs [22, 69–71]. Ikpe et al. [72] stated that governance moderates the relationship between health costs and economic growth in Sub-Saharan African countries. Rahman et al. [73] found governance as a moderating factor in the relationship between health cost and good health in BRICS. Further, Albitar et al. [74] also found the moderating role of governance mechanisms in sustainability and firm performance. As the institutional theory supports that governance can enhance health outcomes, there may be a possible interaction of health expenditure and governance towards health outcomes.

### Theoretical framework

Institutional Theory, originally developed by sociologists such as James March and Johan Olsen, and further expanded by scholars like W. Richard Scott and Douglass North, focuses on the role of institutions in shaping organisational behaviour and societal outcomes [36–38]. This theory defines institutions as established norms, rules, and structures that guide and constrain human behaviour within various contexts [34]. According to Institutional Theory, these formal and informal institutions influence how resources are allocated and utilised, impacting overall effectiveness and outcomes [34]. In the context of health care expenditure and its impact on good health and well-being, Institutional Theory suggests that the effectiveness of health investments is significantly influenced by the quality of governance and institutional frameworks [34, 39]. The theory posits that in well-governed systems with strong institutions, health expenditure is more likely to be effectively managed and directed toward impactful health interventions, resulting in better health outcomes and improved SDG3 ratings. Conversely, in systems with weaker institutions, health expenditures may not translate into significant improvements due to issues such as misallocation, inefficiency, and corruption [75, 76].

**Fig. 1:**
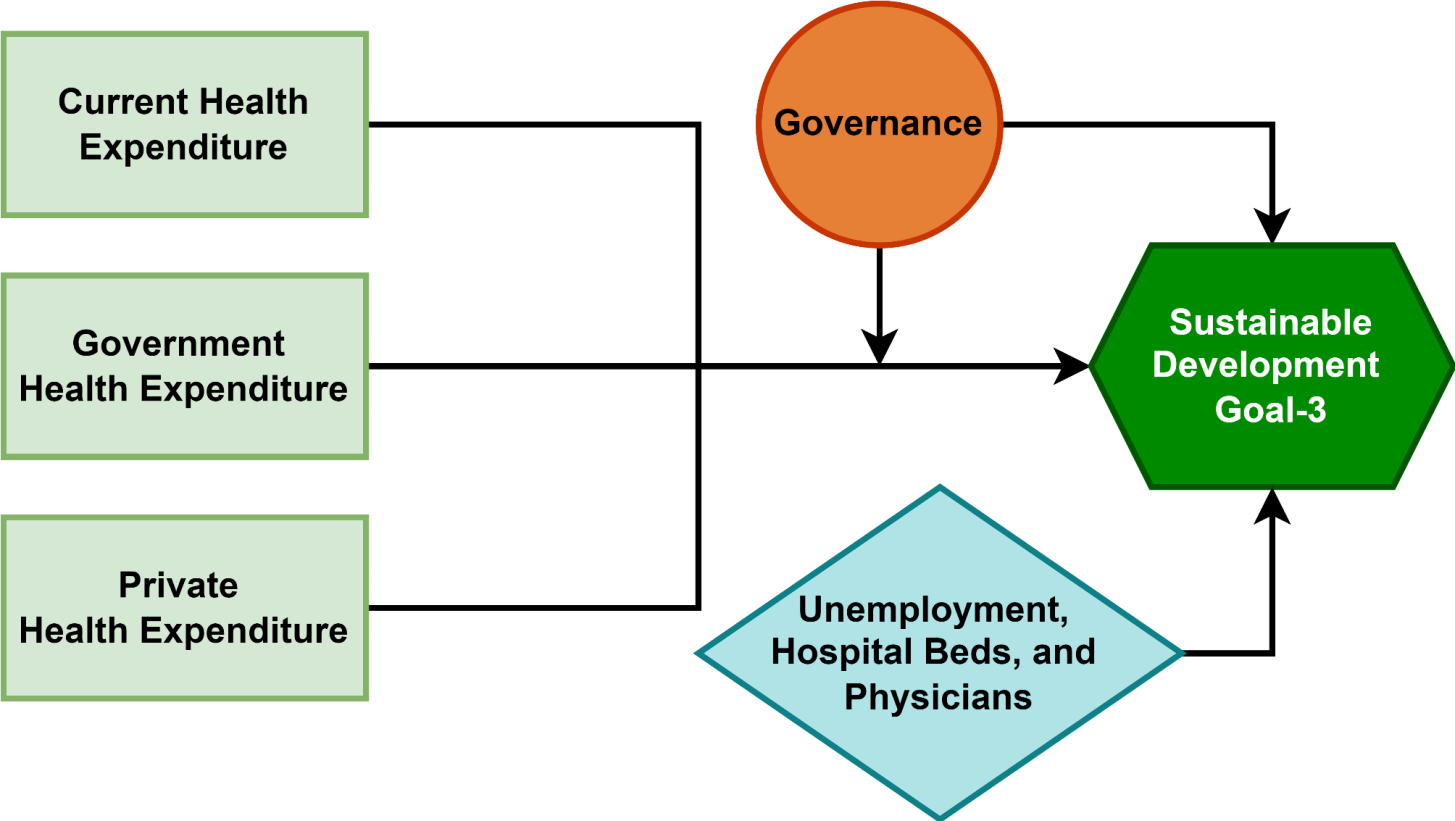
Conceptual framework developed by the authors

According to North [77], institutions serve as the rules of the game that shape economic and social interactions, influencing the incentives and behaviours of individuals and organisations. In the context of this study, governance, as a critical institutional factor, can moderate the relationship between health expenditure and the achievement of SDG 3 by varied levels of governance in which resources are allocated, managed, and monitored. Figure 1 illustrates the conceptual framework for this study, designed to explore the impact of health expenditure on health outcomes within the context of Institutional Theory. In this framework, current health expenditure (CHE), domestic government health expenditure (DGHE), and domestic private health expenditure (DPHE) serve as independent variables, representing different sources of investment in the health sector. Then, governance serves as an independent variable the same way health expenditure does. Additionally, as institution which has a direct bearing on the economic environment upon which productive activity takes place to determine outcomes, governance serves as a catalyst in the relationship between health expenditure and SDG3. This variable captures the quality of institutions, which are expected to strengthen the positive effects of health expenditure on health outcomes. Control Variables including unemployment, number of hospital beds, and number of physicians, are incorporated to account for other factors that could influence health outcomes.

## Methodology

### Data and sample

This study employs panel data from the BRICS economies—Brazil, Russia, India, China, and South Africa—over the period from 2000 to 2023. These countries were selected due to their growing influence as emerging economies and the considerable diversity in their governance systems and health expenditure patterns [1, 22, 69, 70]. The BRICS economies offer a compelling context for studying the interaction between governance and health investments, particularly in achieving SDG 3, which emphasises good health and well-being [22, 69]. The data for this study is sourced from reputable databases, such as the World Development Indicators (WDI), Worldwide Governance Indicators [78] for economic, governance, and health-related variables and the Sustainable Development Goals Index (SDGI) for measuring SDG3. By focusing on BRICS, the study can analyse a wide spectrum of governance quality, health expenditure structures, and socioeconomic factors, making these economies ideal for understanding how good governance interacts with SDG3. Table 1 shows the data with sources.

**Table 1:** Data source and variables

Variables	Sign	Expected Relationships	Measurement/description	Sources
Good health and Well-being	SDG3		SDG3 is measured using the overall score of the targets in good health and wellbeing.	SDGI
Current health expenditure	CHE	Positive	Current health expenditure (% of GDP)	WDI
Government Health expenditure	DGHE	Positive	Domestic government health expenditure (% of general government expenditure)	WDI
Private Health Expenditure	DPHE	Positive	Domestic private health expenditure (% of current health expenditure)	WDI
Governance	GGOV	Positive	Governance is composite measured using six indicators: voice and accountability, political stability and absence of violence/terrorism, government effectiveness, regulatory quality, rule of law, and control of corruption. Principal component analysis (PCA) was used to get the value of GGOV.	[78]
Unemployment	UNEM	Negative	Unemployment, total (% of the total labour force)	WDI
Number of Hospital Beds	NHB	Positive	Hospital beds (per 1,000 people)	WDI
Number of Physicians	NPH	Positive	Physicians (per 1,000 people)	WDI

### Variables

The variables used in this study are categorised into dependent, independent, moderating, and control variables, each selected based on their theoretical and empirical relevance to the research question.

SDG3: The dependent variable is SDG3, which measures the progress of each country toward achieving the health-related goals set by the United Nations [3]. A higher SDG3 score reflects better health outcomes, such as reduced mortality rates, improved access to healthcare, and overall well-being [3]. This variable captures the effectiveness of health policies and expenditures in improving public health outcomes. The greater the SDG3 score, the better the indication for health outcomes.

Current Health Expenditure (CHE): CHE is measured as a percentage of GDP, and captures total health expenditure by both the public and private sectors [6, 71]. A positive relationship with SDG3 is expected, as higher health spending generally leads to improved health infrastructure and services.

Domestic Government Health Expenditure (DGHE): DGHE represents the share of government spending on health as a percentage of total government expenditure [26]. This variable is expected to have a strong positive impact on health outcomes, given that government health spending typically targets essential services like hospitals, public health campaigns, and vaccinations.

Domestic Private Health Expenditure (DPHE): DPHE captures the share of private sector health expenditure [5]. While private spending may improve access to healthcare for some, the relationship with SDG3 could vary depending on how equitably these resources are distributed.

**Fig. 2:**
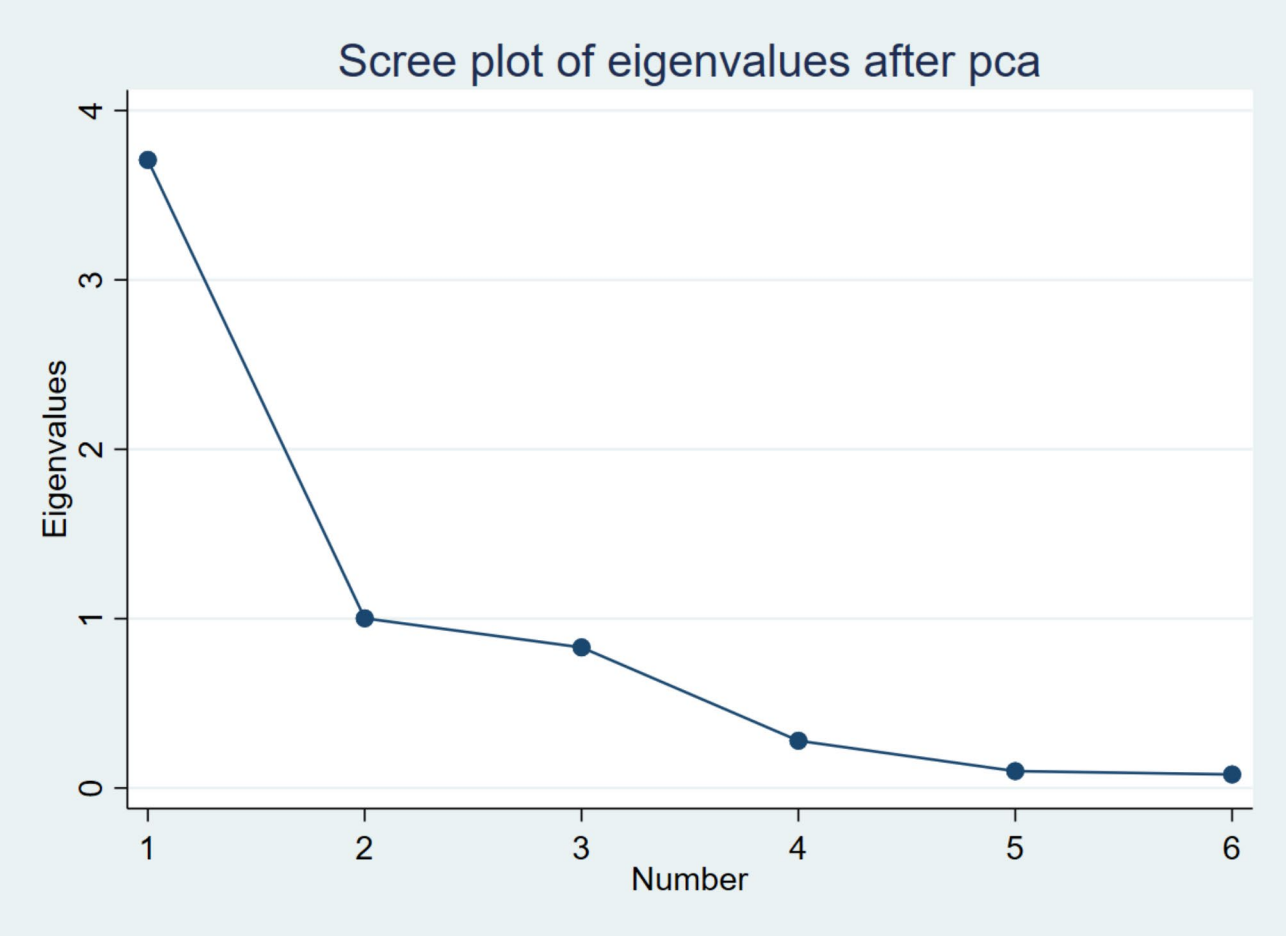
Scree plot of eigenvalues after PCA for governance variable

GGOV: The interaction variable is Governance (GGOV), which is measured using six key indicators: voice and accountability, political stability, government effectiveness, regulatory quality, rule of law, and control of corruption [35]. Kaufmann et al. [25] showed the governance variables with six indicators. A higher governance score is expected to strengthen the positive effects of health expenditure on SDG3, as good governance ensures the efficient allocation of resources, reduces corruption and enhances policy implementation. The interaction between governance and health expenditure is central to this study, as strong governance is likely to amplify the benefits of health spending on public health outcomes. PCA was employed to calculate the governance variable to ensure a robust and composite measure of institutions following Wang [79] and Ndzignat Mouteyica and Ngepah [80]. The principal component analysis is the widely used statistical technique to retain the most significant variance across indicators by reducing the dimensionality of data. The use of PCA ensures the reflection of the combined impact of the six indicators of governance on health outcomes (see Fig. 2). In the interaction, the marginal effect analysis was employed to check the marginal effect of health expenditure on health outcomes with varied levels (Minimum, Average, and Maximum level) of governance following the concept of Ikpe et al. [72], Tchamyou et al. [81], Slesman et al. [82], and Slesman et al. [83]. Minimum, average, and maximum levels indicate the weak, moderate, and good level of governance, respectively.

In addition, the study includes several control variables that could influence SDG3. Unemployment (UNEM): UNEM is measured as a percentage of the total labour force, and is expected to have a negative impact on health outcomes, as economic stress from unemployment often leads to reduced access to healthcare.

Number of Hospital Beds (NHB): NHB includes inpatient beds available in public, private, general, and specialised hospitals and rehabilitation centres. In most cases, beds for both acute and chronic care are included.

Number of Physicians (NPH): NPH refers to licensed medical doctors who are highly trained to diagnose and treat illnesses, prescribe medications, and perform medical procedures, typically working in hospitals or clinics.

### Model specification

To assess the relationship between health expenditure, governance, and SDG3 outcomes, econometric models are specified. The baseline model examines the direct effect of health expenditures (CHE, DGHE, DPHE), governance (GGOV), and control variables on SDG3 outcomes. The baseline model is specified as following Huang et al. [84]:

1.1 $$\eqalign{{SDG3}_{it}&=C+\:{\beta\:}_{1}{CHE}_{it}+\:{\beta\:}_{2}{GGOV}_{it}+\:{\beta\:}_{3}{UNEM}_{it} \cr &+\:{\beta\:}_{4}{NHB}_{it}+\:{\beta\:}_{5}{NPH}_{it}+\:\:{\in\:}_{it}}$$

1.2 $$\eqalign{{SDG3}_{it}& =C+\:{\beta\:}_{1}{DGHE}_{it}+\:{\beta\:}_{2}{GGOV}_{it}+\:{\beta\:}_{3}{UNEM}_{it}\cr & +\:{\beta\:}_{4}{NHB}_{it}+\:{\beta\:}_{5}{NPH}_{it}+\:\:{\in\:}_{it}}$$

1.3 $$\eqalign{{SDG3}_{it} & =C+\:{\beta\:}_{1}{DPHE}_{it}+\:{\beta\:}_{2}{GGOV}_{it}+\:{\beta\:}_{3}{UNEM}_{it} \cr & +\:{\beta\:}_{4}{NHB}_{it}+\:{\beta\:}_{5}{NPH}_{it}+\:\:{\in\:}_{it}}$$

In addition to the baseline model, interaction models are used to explore how governance interacts with the effect of health expenditure on SDG3 outcomes. Interaction Model A tests the moderating effect of governance on current health expenditure (CHE). The interaction term between GGOV and CHE is included to assess whether better governance enhances the effectiveness of CHE in improving health outcomes. The Model A is specified by Eq. 2 as:

2 $$\eqalign{{SDG3}_{it}& =C+\:{\beta\:}_{1}{CHE}_{it}+{\beta\:}_{2}{GGOV\times\:\text{C}\text{H}\text{E}}_{it}+\:{\beta\:}_{3}{UNEM}_{it}\cr & +\:{\beta\:}_{4}{NHB}_{it}+\:{\beta\:}_{5}{NPH}_{it}+\:{\in\:}_{it}}$$

Interaction Model B examines the interaction between governance and domestic government health expenditure (DGHE). This model tests whether strong governance improves the allocation and impact of government health spending. Model B is specified by Eq. 3 as:

3 $$\eqalign{{SDG3}_{it} & =C+\:{\beta\:}_{1}{DGHE}_{it}+{\beta\:}_{2}{GGOV\times\:\text{D}\text{G}\text{H}\text{E}}_{it} \cr & +\:{\beta\:}_{3}{UNEM}_{it}+\:{\beta\:}_{4}{NHB}_{it}+\:{\beta\:}_{5}{NPH}_{it}+\:{\in\:}_{it}}$$

Finally, Interaction Model C assesses the interaction effect of governance on domestic private health expenditure (DPHE), testing whether governance can enhance the effectiveness of private health spending. This Model C is specified by Eq. 4 as:

4 $$\eqalign{{SDG3}_{it} & =C+\:{\beta\:}_{1}{DPHE}_{it}+{\beta\:}_{2}{GGOV\times\:\text{D}\text{P}\text{H}\text{E}}_{it}\cr & +\:{\beta\:}_{3}{UNEM}_{it}+\:{\beta\:}_{4}{NHB}_{it}+\:{\beta\:}_{5}{NPH}_{it}+\:{\in\:}_{it}}$$

Where, SDG3 = sustainable development goal 3: health and well-being, CHE = current health expenditure, DGHE = domestic government health expenditure, DPHE = domestic private health expenditure, GGOV = good governance, UNEM = unemployment, ECCGR = economic growth, NHB = number of hospital beds, NPH = number of physicians, NHW = number of health workers, C = Constant, $\:\times\:$= multiplication that indicates interaction of variables, and $\:\in\:$ = error term.

This study employs FMOLS that deals with issues of serial correlation and heteroskedasticity and also adjusts for endogeneity, ensuring efficient and unbiased long-term estimates [85]. However, the system GMM was employed to ensure the correct use of instrumental variables for the causation of health expenditure, governance, and SDG3 following Slesman et al. [82] and Huang et al. [84].

Before estimating the models, several diagnostic tests are conducted to ensure the validity of the results. First, the Cross-Sectional Dependence (CSD) Test is applied to account for possible interdependencies between the BRICS economies, given their economic and geopolitical connections [86]. Ignoring cross-sectional dependence could lead to biased estimates. Second, stationarity tests such as the Levin-Lin-Chu or Im-Pesaran-Shin tests are performed to ensure that the variables are not non-stationary, which could otherwise lead to spurious results [12, 87]. Lastly, cointegration tests are conducted to verify whether the variables share a long-run equilibrium relationship, which justifies the use of FMOLS for long-term estimation [12, 88, 89]. Finally, the marginal effect analysis was employed for the different levels of governance following Ikpe et al. [72] and Slesman et al. [83].

## Results

### Descriptive statistics and correlations

The mean, standard deviation, and correlation matrix for the variables are shown in Table 2 to show their central tendencies, variability, and relationships. The sample period’s mean health outcomes, expenditures, governance quality, and related characteristics are shown. SDG3’s mean score is 67.5, representing overall health results, while CHE averages 5.2% of GDP and DGHE 14.8% of total government expenditure. The standard deviations demonstrate the variability around these averages, with variables like DPHE showing significant variability. With correlations below 0.80, the correlation matrix shows that health expenditures, outcomes, and governance quality are positively correlated, but multicollinearity is not an issue [90]. The greatest correlation between SDG3 and GGOV is 0.72, significantly below the 0.80 criterion. The low correlation among independent variables reduces the probability of multicollinearity impacting calculated coefficients, making regression models robust.

**Table 2:** Descriptive statistics and correlations of the investigated variables

Variables	(1)	(2)	(3)	(4)	(5)	(6)	(7)	(8)
(1) SDG3	1.000							
(2) CHE	0.113	1.000						
(3) DGHE	0.008	0.724	1.000					
(4) DPHE	-0.298	-0.378	-0.788	1.000				
(5) GGOV	-0.487	0.100	-0.184	0.505	1.000			
(6) UNEM	-0.620	0.548	0.614	-0.230	0.432	1.000		
(7) NHB	0.305	-0.099	0.212	-0.627	-0.804	-0.283	1.000	
(8) NPH	0.541	0.037	0.205	-0.613	-0.850	-0.377	0.738	1.000
Mean	64.504	5.99	9.047	53.278	0	10.709	3.649	1.835
Std. Dev.	14.615	2.011	3.779	13.881	1	7.898	2.652	1.391
Minimum	34.164	2.86	2.65	28.06	-2.298	3.076	0.49	0.237
Maximum	84.018	10.182	17.72	79.78	1.409	34.007	10.72	4.997

### Cross-sectional dependency, stationarity, and cointegration tests

Table 3 shows Pesaran’s Cross-Sectional Dependence (CSD) test findings for research variables. CSD tests determine if variables are cross-sectionally dependent, which can affect panel data analysis validity [88]. The CD statistic and *p*-value for each variable are shown in the table. The results reject the null hypothesis of no cross-sectional dependence for all variables. These variables may be influenced by comparable factors across cross-sectional units [89]. Most variables have strong cross-sectional dependence, indicating that observations across cross-sections are not independent, stressing the significance of accounting for this reliance in the analysis to minimise bias.

**Table 3:** CSD tests

Variables	Pesaran CD Statistic	*p*-value	Interpretation
SDG3	14.613	0.000	Reject the null of no cross-sectional dependence.
CHE	3.508	0.000	
DGHE	8.243	0.000	
DPHE	8.868	0.000	
GGOV	6.374	0.000	
UNEM	10.129	0.000	
NHB	19.659	0.000	
NPH	3.510	0.000	

Stationarity must be tested before regression analysis on time-series or panel data to avoid misleading findings [87]. The Levin, Lin & Chu (LLC) and Im, Pesaran, and Shin (IPS) tests were used to analyse variable stationarity in this 2000–2023 BRICS study [85]. Null hypothesis: panel data has a unit root, indicating non-stationarity; alternative hypothesis: data is stationary. Table 4 shows all variables pass stationarity tests at level [I(0)] and at first difference [I(1)].

**Table 4:** Stationarity tests

Variable	LLC Test Statistic	IPS Test Statistic	Stationarity
SDG3	-2.280*	4.265***	I (0)
CHE	-7.421***	-3.848***	I (1)
DGHE	-8.566***	-4.155***	I (1)
DPHE	-4.238**	-3.622***	I (0)
GGOV	-8.730***	-5.052***	I (1)
UNEM	-8.343***	-4.478***	I (1)
NHB	-2.372**	-3.784***	I (0)
NPH	-6.054***	-5.797***	I (1)

Note: ***=*p* < 0.01, **=*p* < 0.05, and *=*p* < 0.10

The cointegration test was employed to check the long-term equilibrium relationship despite short-term volatility as stationarity is confirmed [89]. Table 5 reveals the results of the cointegration tests conducted using both the Pedroni and Kao methods. The Pedroni Cointegration Test yields mixed results: the Panel PP-Statistic, Panel ADF-Statistic, and Group PP-Statistic show significant cointegration. The variables appear to be in long-term equilibrium [12]. The Panel and Group rho-statistics do not support cointegration but the Kao Residual Cointegration Test shows long-run cointegration between the variables [85]. This validates the use of long-run estimate methods like FMOLS in the following investigations, which account for long-term relationships and assure that short-term deviations will correct themselves.

**Table 5:** Cointegration tests employing Pedroni and Kao tests

Statistic	Test Value	*p*-value	Decision
***Pedroni Cointegration Test***:			
Panel v-Statistic	2.10	0.0175	Reject H₀
Panel rho-Statistic	-1.56	0.0590	Do not reject H₀
Panel PP-Statistic	-2.45	0.0072	Reject H₀
Panel ADF-Statistic	-2.98	0.0029	Reject H₀
Group rho-Statistic	-0.65	0.2572	Do not reject H₀
Group PP-Statistic	-3.21	0.0013	Reject H₀
Group ADF-Statistic	-3.08	0.0021	Reject H₀
***Kao Residual Cointegration Test***:			
Kao Residual Test	-1.898	0.0288	Reject H₀

### FMOLS estimation results

This study used FMOLS estimation for the baseline and interaction models (A, B, and C). The FMOLS is employed as the series are confirmed stationary, and cointegrated in the long run. FMOLS accounts for serial correlation in the error terms and endogeneity (correlation between the error term and regressors) that can arise in the cointegrated systems. Further, FMOLS produces reliable estimates even with small sample sizes. Therefore, this study selects FMOLS over ordinary least square. Table 6 shows the results where CHE, DGHE, and DPHE are considered health expenditure variables that have an impact on SDG3. According to the baseline model, current health expenditure and government health expenditure positively affect SDG3 outcomes while private health expenditure has a negative impact on SDG3. This study found that governance has also a positive impact on SDG3.

According to Model-A which includes the interaction term *CHE × GGOV*, this study found that the relationship between current health expenditure and SDG3 is positively moderated by governance. Also, the results of *DGHE × GGOV* indicate the positive moderation effect in Model-B. Further, Model-C (*DPHE × GGOV*) reveal that the relationship between domestic private health expenditure and SDG3 is negatively affected by governance. These three models assess how the relationship between government health spending and SDG3 is influenced by the quality of governance. Thus, the interaction effect highlights that governance not only contributes directly to better health outcomes but also enhances the impact of government health investments. These results reinforce the critical role of governance in optimizing the effectiveness of health expenditure policies and achieving better health outcomes.

On the other hand, this study found unemployment negatively causes SDG3, which aligns with the expectation that higher unemployment negatively impacts health outcomes. Similarly, the number of hospital beds negatively affects SDG3 outcomes. Finally, for the number of physicians, this study found a positive impact on SDG3. Thus, the more the number of physicians, the better the health outcomes.

**Table 6:** FMOLS estimation results

	Baseline Model			Model-A	Model-B	Model-C
	SDG3	SDG3	SDG3	SDG3	SDG3	SDG3
CHE	3.920**			4.104**		
	(0.2167)			(0.2089)		
	[18.0904]			[19.6456]		
CHE*GGOV				0.026*		
				(0.0609)		
				[0.4310]		
DGHE		2.860**			3.163**	
		(0.0958)			(0.1012)	
		[29.8558]			[31.2709]	
DGHE*GGOV					0.335**	
					(0.0405)	
					[8.2658]	
DPHE			-0.677**			-0.675**
			(0.0201)			(0.0203)
			[-33.7085]			[-33.2743]
DPHE*GGOV						0.028**
						(0.0083)
						[3.3520]
GGOV	2.148**	3.794**	1.956			
	(0.4336)	(0.4735)	(0.4639)			
	[4.9541]	[8.0115]	[4.2170]			
UNEM	0.043	0.098	0.257**	-0.076	0.012	0.237**
	-0.077	-0.081	(0.0776)	-0.073	-0.076	(0.0780)
	[0.5548]	[1.2174]	[3.3100]	[-1.0464]	[0.1527]	[3.0401]
NHB	-4.311**	-4.985**	-4.541**	-4.089**	-4.448**	-4.634**
	(0.2446)	(0.2730)	(0.2652)	(0.2360)	(0.2637)	(0.2758)
	[-17.6250]	[-18.2613]	[-17.1201]	[-17.3250]	[-16.8691]	[-16.8041]
NPH	9.851**	12.609**	6.135**	10.419**	11.201**	6.692**
	(0.5958)	(0.6715)	(0.6396)	(0.5960)	(0.7131)	(0.6294)
	[16.5327]	[18.7769]	[9.5922]	[17.4824]	[15.7070]	[10.6321]
R-squared:	0.849	0.905	0.927	0.848	0.904	0.926

Note: SDG3 is the dependent variable. CHE, DGHE, and DPHE are the health expenditure variables. GGOV is the interaction variable. ***=*p* < 0.01, **=*p* < 0.05, and *=*p* < 0.10. The first bracket includes robust standard errors, and the third bracket includes t-statistics.

### Robustness check using alternative estimators

To ensure the robustness and reliability of the findings from the FMOLS estimations, this study conducted an additional round of analysis using the system GMM. Robustness checks are essential because they confirm that the results are not sensitive to the choice of estimation method, thereby strengthening the validity of the conclusions drawn [87]. According to Table 7, current health expenditure and government health expenditure positively influence SDG3 but private health expenditure negatively influences SDG3. Further, the interaction term *CHE × GGOV* positively affects SDG3 but not statistically significant; and *DGHE × GGOV* shows positive and statistically significant impact on SDG3. But *DPHE × GGOV* shows positive and insignificant effect on SDG3. The results of the system GMM estimation are consistent with the FMOLS across all models. This consistency in results enhances the robustness and credibility of the conclusions drawn about the relationships between health expenditures, governance, and health outcomes. The system GMM is employed for robustness checks as it effectively mitigates endogeneity, omitted variable bias, and measurement errors, thereby ensuring the validity of dynamic panel data estimations [82, 84]. The approach utilises instruments, thereby improving efficiency and reliability in identifying causal relationships. It also addresses unobserved heterogeneity and considers potential biases resulting from fixed effects or weak instruments.

**Table 7:** Robustness check using system GMM

	Baseline Model			Model-A	Model-B	Model-C
	SDG3	SDG3	SDG3	SDG3	SDG3	SDG3
SDG3 (-1)	0.981***	0.967***	0.949***	0.985***	0.970***	0.942***
	[0.0107]	[0.0149]	[0.0124]	[0.0052]	[0.0085]	[0.0106]
CHE	0.221**			0.193***		
	[0.0107]			[0.0313]		
*CHE × GGOV*				0.062		
				[0.0407]		
DGHE		0.015			0.020	
		[0.0270]			[0.0185]	
*DGHE × GGOV*					0.044**	
					[0.0213]	
DPHE			-0.031***			-0.037***
			[0.0114]			[0.0115]
*DPHE × GGOV*						0.008
						[0.0069]
GGOV	0.095	0.024	0.046	-0.397	0.488*	0.411
	[0.1228]	[0.1081]	[0.1130]	[0.2451]	[0.2808]	[0.3175]
UNEM	0.014	-0.044**	-0.077***	-0.006	-0.057***	-0.095***
	[0.0281]	[0.0206]	[0.0161]	[0.0102]	[0.0148]	[0.0.0142]
NHB	-0.319***	-0.189	-0.384***	-0.261**	-0.106	-0.383***
	[0.1121]	[0.1560]	[0.0764]	[0.1239]	[0.1689]	[0.1438]
NPH	0.701**	0.353	0.566***	0.459*	0.094	0.540*
	[0.2801]	[0.3575]	[0.2350]	[0.2610]	[0.3764]	[0.2971]
Constant (c)	3.0087***	3.239***	6.822***	2.938***	3.3696***	7.926***
	[0.5109]	[1.0862]	[1.1182]	[0.3124]	[0.6168]	[1.1531]
Sargan test (*p*-value)	0.207	0.016	0.110	0.145	0.900	0.161
Hansen J-Test (*p*-value)	1.000	1.000	1.000	1.000	1.000	1.000
AR(1) Test (*p*-value)	0.035	0.035	0.036	0.034	0.034	0.035
AR(2) Test (*p*-value)	0.562	0.554	0.543	0.562	0.562	0.544
Instrument ratio i/j	0.575	0.575	0.575	0.958	0.958	0.958

Note: SDG3 is the dependent variable. CHE, DGHE, and DPHE are the health expenditure variables. GGOV is the interaction variable. The null hypothesis of the Hansen J-test indicates the instruments are valid while the Arellano-Bond (AR-2) test’s null hypothesis refers to the absence of second-order autocorrelation in the residuals. Standard errors are in the parentheses. ***=*p* < 0.01, **=*p* < 0.05, and *=*p* < 0.10

### Robustness check using alternative governance measure

This study introduced a new variable governance dummy (GVD). Value 1 is used if the yearly index is greater than the mean value indicating the high or good governance, otherwise, 0, indicates the low governance. This study replaced the continuous form of governance variable by this GVD. Table 8 shows the results of the final estimations presented in Table 6. According to Table 8, the results are consistent, particularly, CHE and DGHE positively affect SDG3 outcomes, while DPHE negatively affects SDG3. The results of interaction effects are also consistent indicating the better robustness of the key findings.

**Table 8:** Robustness check using governance dummy

	Baseline Model			Model-A	Model-B	Model-C
	SDG3	SDG3	SDG3	SDG3	SDG3	SDG3
CHE	3.59***			3.737***		
	(0.938)			(0.966)		
CHE*×*GVD				0.328		
				(0.253)		
DGHE		2.76***			2.654***	
		(0.293)			(0.3)	
DGHE*×*GVD					0.397***	
					(0.141)	
DPHE			− 0.645***			− 0.657***
			(0.055)			(0.053)
DPHE*×*GVD						0.029
						(0.024)
GVD	3.878**	3.63***	1.395			
	(1.696)	(1.32)	(1.225)			
UNEM	0.037	− 0.029	0.12	− 0.061	− 0.083	0.13
	(0.307)	(0.233)	(0.207)	(0.307)	(0.23)	(0.208)
NHB	-4.205***	-4.605***	-4.388***	-4.141***	-4.405***	-4.481***
	(0.957)	(0.758)	(0.682)	(0.973)	(0.762)	(0.685)
NPH	9.583***	12.478***	6.547***	10.41***	12.06***	6.723***
	(2.497)	(2.007)	(1.759)	(2.521)	(2.051)	(1.722)
Constant	37.746***	31.292***	100.632***	37.438***	32.957***	101.053***
	(6.201)	(3.954)	(4.543)	(6.362)	(4.009)	(4.438)
R-squared	0.323	0.575	0.656	0.301	0.577	0.656

Standard errors are in parentheses. *** *p* < 0.01, ** *p* < 0.05, * *p* < 0.1

### Marginal effects of health expenditure at different levels of governance

This study found that governance interacts with the relationship between health expenditure and SDG3 outcomes in the net effects. The marginal effect of the health expenditure from the interaction coefficient must be analysed from Eq. 2, 3, and 4 as follows:

5 $$\:\frac{dY}{dX}={\beta\:}_{1}+\:{\beta\:}_{3}M,$$

**Fig. 3:**
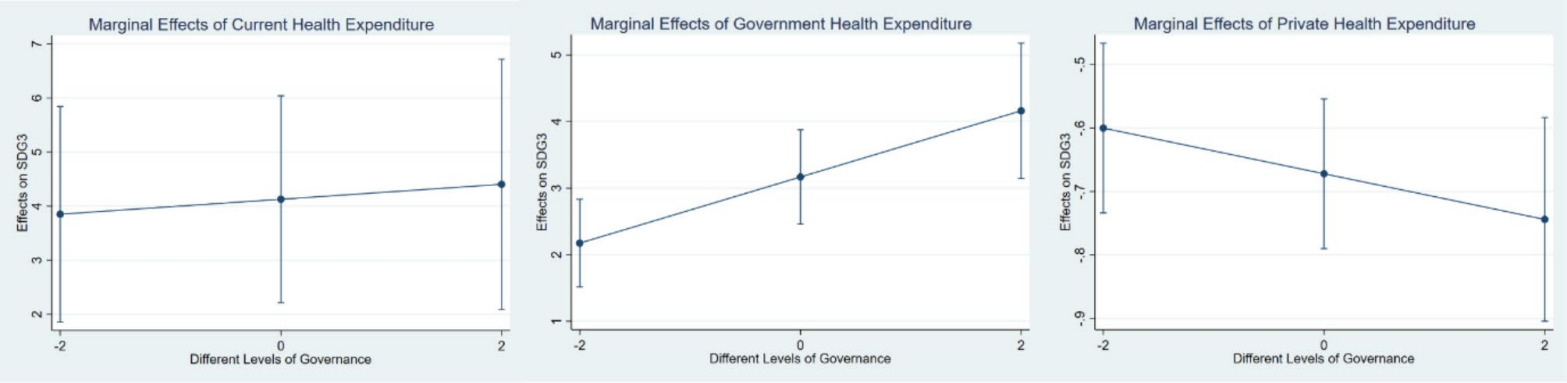
Marginal effects of health cost at different levels of governance

**Fig. 4:**
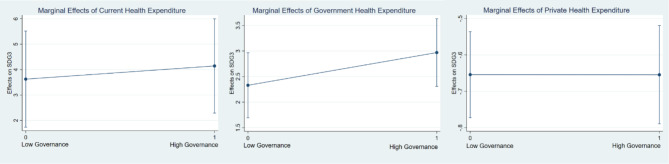
Marginal effects of health cost at governance dummy

Where, Y indicates SDG3, X indicates CHE, DGHE, and DPHE, M is the GGOV, $\:{\beta\:}_{1}$ indicates the main effect of X, and $\:{\beta\:}_{3}$ is the interaction effect between X and M. If the values of $\:{\beta\:}_{3}$ > 0, it indicates that governance enhances the relationship between health expenditure and SDG3 outcomes; $\:{\beta\:}_{3}$ <0 = Weakens the relationship; and $\:{\beta\:}_{3}$=0 indicating there is no interaction effect. The marginal effects analysis indicates that governance substantially interacts with the relationship between health expenditure and SDG3 outcomes. The positive impact of current and government health expenditure on health outcomes is comparatively weaker at lower levels of governance (Model-A and B), but private health expenditure and SDG3 outcomes are weakening by governance at different levels which is indicative of inefficiencies in resource allocation and implementation (see Table 9). Further, this study checked the robustness of the marginal effects using the governance dummy variable in Table 10. The results are also consistent. Figures 3 and 4 shows the visualisations of the marginal effects. The marginal effect enhances as governance improves to mean or average levels, suggesting that improved governance enables the effective utilisation of health expenditure to improve health outcomes. The marginal effect reaches its maximum at the highest level of governance, indicating that good governance enhances the impact of health expenditure by guaranteeing transparency, accountability, and optimal resource distribution. These results underscore the indispensable function of governance in optimizing the efficacy of health expenditures in the context of sustainable development.

**Table 9:** Marginal effect of health expenditure at different levels of governance

Marginal Effects	Different levels of Governance		
	Minimum	Average	Maximum
Model-A: Marginal effect of CHE at GGOV	3.851***	4.127***	4.403***
	(1.016)	(0.977)	(1.180)
Model-B: Marginal effect of DGHE at GGOV	2.174***	3.168***	4.162***
	(0.336)	(0.361)	(0.518)
Model-C: Marginal effect of DPHE at GGOV	-0.600***	-0.672***	-0.744***
	(0.068)	(0.060)	(0.082)

***=*p* < 0.01. Standard errors are in the parentheses

**Table 10:** Marginal effect of health expenditure using governance dummy

Marginal Effects	Levels of Governance	
	Low Governance (0)	High Governance (1)
Model-A: Marginal effect of CHE at GVD	3.625***	4.143***
	(0.964)	(0.945)
Model-B: Marginal effect of DGHE at GVD	2.330***	2.969***
	(0.324)	(0.337)
Model-C: Marginal effect of DPHE at GVD	-0.654***	-0.655***
	(0.060)	(0.069)

***=*p* < 0.01. Standard errors are in the parentheses

## Discussions of findings

Results from different estimations in this study show that government and current health spending show positive effects but private health expenditure shows a negative impact on health outcomes in the economies of the BRICS. Particularly, 1% increase in current health expenditure and government health expenditure leads to positive enhancement of SDG3 by 3.920% and 2.860%, respectively. However, 1% increase in private health expenditure negatively reduces SDG3 by 0.677%. The reasons for negative impact of private health expenditure in BRICS nations due to the market failures in private healthcare occur because profit-oriented models emphasise expensive treatments rather than accessible preventive care, resulting in inequitable access, particularly in rural regions. Trade-offs between quality and accessibility are apparent since private healthcare institutions mostly serve affluent people, but lower-income groups encounter financial obstacles, frequently postponing or forgoing essential treatments. Health system fragmentation is notably evident in nations such as India and Brazil, where concurrent public and private healthcare systems generate differences in service quality and accessibility, hence constraining overall health advancements. Enhancing public healthcare investments and regulatory frameworks is essential for addressing these challenges and ensuring fair advancement toward SDG3. This is partially consistent with the findings of Ahmad and Hasan [63], who noted that public health expenditure enhances health outcomes in Malaysia. Banik et al. [4] demonstrated a robust correlation between health expenditure and human development, highlighting the essential importance of financial investments in enhancing social well-being. Ewurum [7] revealed similar findings in Nigeria, indicating that public health expenditure improves health status. Ganda [69] offered an opposing viewpoint by demonstrating that health expenditure affects environmental quality, indicating that the results of such investments may transcend direct health measurements and encompass wider sustainability objectives.

This study also found that 1% increase in governance positively improves SDG3 outcomes by 2.148%, a finding consistent with Hilaire [24], who highlighted the role of good governance in improving public health expenditure effectiveness in Africa. Kirigia and Kirigia [30] emphasised the essential role of governance in health development, while Ndzignat Mouteyica and Ngepah [80] demonstrated the importance of governance in ensuring convergence in health outcomes across African nations. Finally, this study reveals that 1% changes in the current health expenditure-governance interaction term lead to positively enhanced SDG3 by 0.026%, where marginal effect coefficients at the minimum, average, and maximum level of governance are greater than zero indicating that governance positively enhances the relationship between current health expenditure and SDG3. Additionally, 1% increase in the government health expenditure-governance interaction term leads to a positively enhance SDG3 by 0.335%, where marginal effect coefficients at different levels of governance are greater than zero, indicating that governance positively enhances the relationship between government health expenditure and SDG3. However, 1% changes in private health expenditure-governance interaction term leads to a decrease in SDG3 by 0.028%, where the marginal effect coefficients at different levels of governance are less than zero, indicating that governance decreases the impact of private health expenditure on SDG3. These findings complement prior studies, such as those by Liu et al. [91], Ndzignat Mouteyica and Ngepah [80], Sahoo et al. [21], Wang [79], Hilaire [24], Farag et al. [42], and Ewurum [7], which collectively emphasise the critical interplay between governance and health expenditure. Our study extends this discourse by offering empirical evidence on the moderating role of governance in the BRICS economies, providing actionable insights for policymakers aiming to achieve SDG3.

Good governance ensures the efficient allocation of resources, transparency, and accountability, which are essential for maximizing the impact of health spending [42, 63]. Institutions play a pivotal role in this dynamic, as institutional theory suggests that well-established governance structures create an enabling environment for effective policy implementation and resource utilisation [34]. Therefore, good regulatory frameworks and anti-corruption measures can enhance the quality and reach of healthcare services, particularly in regions with historically weak governance. Drawing from the findings of Hilaire [24] and Ndzignat Mouteyica and Ngepah [80], governance quality can address barriers such as inefficiencies in health systems, inequitable access, and poor service delivery. Policies that strengthen institutional capacity—such as training for health administrators, digital governance tools, and participatory policymaking—can further amplify the benefits of health expenditure. Rahman et al. [14] and Makuta and O’Hare [92] support the institutional theory by demonstrating that governance quality significantly enhances the efficiency of public health spending, leading to better health outcomes. In contrast, Filmer and Pritchett [41] challenge this perspective, arguing that in low-governance settings, increased health expenditure does not necessarily translate into improved health outcomes due to institutional inefficiencies.

Globalisation facilitates access to international funding, advanced technologies, and global best practices, which, when coupled with robust governance, significantly amplify the impact of health investments [93, 94]. For instance, development-oriented health expenditures (DGHE), often supported by global partnerships, demonstrate stronger effects on health outcomes under conditions of good governance, reflecting the critical interplay between local institutions and global resources [95]. At the same time, globalisation introduces vulnerabilities, such as economic dependencies and exposure to global health crises, which demand resilient governance systems to mitigate risks and ensure equitable resource distribution [96]. The findings highlight that good governance not only optimises domestic health spending but also enhances the capacity of nations to harness globalisation’s benefits, transforming external influences into drivers of improved health outcomes and sustainable development [97].

The BRICS countries, as emerging global powers, are increasingly asserting their geopolitical influence across health and economic policy sectors, shaping regional and international agendas [98, 99]. Their collective economic weight and strategic collaborations position them as pivotal actors in addressing transnational health challenges and promoting equitable global health governance [100]. The results of this study underscore the significance of this influence, particularly in how governance enhances the effectiveness of health expenditures. For example, the amplified impact of development-oriented health expenditures (DGHE) within strong governance frameworks reflects the potential of BRICS nations to lead by example in aligning health investments with institutional reforms [101]. Furthermore, their growing role in multilateral organisations, such as the World Health Organisation and the New Development Bank, allows them to champion policies that prioritise universal health coverage and sustainable financing mechanisms [102]. By leveraging their geopolitical influence, BRICS countries can not only improve domestic health outcomes but also contribute to shaping a more equitable global health and economic order, reinforcing their leadership in achieving SDG3 [103].

## Conclusions and policy implications

This study investigated the impact of health expenditures and governance on health outcomes across BRICS economies from 2000 to 2023, employing dynamic estimation techniques such as FMOLS and system GMM. First, it was established that health expenditures—current, government, and public—positively impact progress toward achieving SDG3 outcomes. Second, governance emerged as a vital determinant of SDG3 outcomes. Finally, the study highlights the interaction effects of governance in the relationship between health expenditure and SDG3. The analysis revealed that the marginal impact of health expenditure on SDG3 varies significantly under different levels of governance—weak, moderate, and good governance, indicating that governance moderates the health cost and SDG3 outcomes. These findings illustrate that governance quality not only enhances the direct effects of health expenditure but also ensures that investments in health translate into tangible outcomes. By showing how governance interacts with health expenditure to improve SDG3, this study complements existing literature [42, 79, 91].

This study’s findings emphasise the necessity for BRICS economies to prioritise heightened investment in health expenditures, especially in governmental and public health spending, to expedite advancement towards SDG3 targets. Policymakers must prioritise the fortification of governance frameworks by improving transparency, accountability, and regulatory standards to guarantee the effective allocation and utilisation of health resources. This can be accomplished by anti-corruption campaigns, capacity-building programmes, and performance-oriented governance structures. Furthermore, promoting regional collaboration within BRICS, as proposed by Liu et al. [91], can enhance knowledge exchange and collective initiatives to tackle common health issues. Governments should adopt a comprehensive approach by integrating institutional reforms with health policy to optimise the synergistic impacts of governance and health expenditures. Ultimately, customised policy responses informed by differing governance quality levels are crucial for achieving equitable health outcomes, especially in areas with deficient governance institutions.

## Limitations of the study and future research path

This study acknowledges the following limitation of this study: this study only focuses on aggregate measures of governance. Analysis using subcomponents of governance would provide a detailed concept of the likely relative important effects of each sub-component of governance on health outcomes. Lastly, the findings are region-specific to the BRICS economies, and although they offer valuable insights, they may not be explicitly generalizable to other regions with distinct economic and institutional contexts. Future research could address these limitations by incorporating country-specific analyses and exploring sub-components to provide a more comprehensive understanding of the interplay between health expenditures, governance, and health outcomes.

## Data Availability

Data for this research was collected from World Development Indicators which is a publicly open-sourced database.
